# Tailoring the Properties of Soy Protein-Based Bioplastics via Plasticizer Composition and Extrusion Temperature for Controlled Iron Release

**DOI:** 10.3390/polym17233209

**Published:** 2025-12-02

**Authors:** Daniel Castro-Criado, Antonio J. Capezza, Alberto Romero, Mercedes Jiménez-Rosado

**Affiliations:** 1 Department of Chemical Engineering, Faculty of Chemistry, University of Seville, 41012 Seville, Spain; 2 Department of Fibre and Polymer Technology, KTH Royal Institute of Technology, SE-100 44 Stockholm, Sweden; ajcv@kth.se; 3 Chemical, Environmental and Bioprocess Engineering Group, I4 Institute, University of León, 24071 León, Spain; mjimr@unileon.es

**Keywords:** soy protein-based matrices, plasticizers, controlled micronutrient delivery, iron release, extrusion processing, biodegradable matrices, sustainable bioplastics

## Abstract

The development of sustainable bioplastic matrices for controlled micronutrient delivery represents a promising strategy in the agri-food and biomedical sectors. This study investigates the influence of plasticizer type (glycerol, water and their mixtures) and processing temperature (70–110 °C) on the fabrication and functional properties of extruded soy protein-based matrices for iron release. Results show that both the nature of the plasticizer and the extrusion temperature critically affect the microstructure and mechanical behavior of the matrices. Specifically, an intermediate glycerol/water ratio (50/50) during extrusion at 90 °C significantly improves matrix resistance, making it optimal for iron-controlled release. These findings underscore the crucial role of formulation and thermal parameters in engineering protein-based delivery systems, thereby paving the way for the design of next-generation biodegradable functional materials.

## 1. Introduction

Since the onset of industrial plastic production in 1950, approximately 8.3 billion tons of plastic have been manufactured [1]. However, the accumulation of plastic waste remains a serious global challenge [2]. Conventional disposal methods, including recycling, landfilling and incineration, offer only partial solutions. Among these, recycling is the most widely implemented due to its ability to reduce CO_2_ emissions and promote material reuse [3]. Nonetheless, achieving sustainable development also demands the creation of new materials that can replace conventional plastics. In this context, biobased and biodegradable materials offer a promising alternative, as they naturally degrade over time and avoid the generation of toxic products [4].

In recent years, bioplastics derived from proteins extracted from agri-food waste have attracted increasing attention [5]. According to the Food and Agriculture Organization (FAO), global agri-food production reached 9.9 billion tonnes in 2023 (which is 990,000 times the weight of the Eiffel Tower), representing a 3% increase from 2022 and a 27% increase since 2010 [6]. Food Loss and Waste (FLW) remains a critical environmental issue. Although rich in nutrients such as proteins, vitamins and minerals, FLW often fails to meet food safety standards and must therefore be discarded [7]. However, this organic waste represents a valuable opportunity for the development of value-added materials. In this context, bio-based polymers are particularly attractive due to their low cost, scalability and minimal environmental impact compared to conventional plastics [8]. Bioplastics have been developed from various renewable sources, including starch [9], pea protein [10], soy protein [11] and sugarcane bagasse [12]. The primary applications of bioplastics include food packaging, biomedical scaffolds, absorbent and superabsorbent materials and agricultural products [13,14]. However, their higher production costs remain a central limit compared to petroleum-derived plastics [15].

Modern agriculture increasingly relies on fertilizers and agrochemicals to enhance crop productivity; however, these inputs often have adverse environmental impacts. For example, volatilization can cause a loss of up to 70% of soil nitrogen, and certain micronutrients may become unavailable to plants due to soil interactions and leaching processes [16]. In this regard, biopolymer matrices derived from agri-food waste offer a sustainable solution for encapsulating nutrients, acting as controlled-release fertilizers (CFRs), thereby preventing premature dissolution or oxidation [17]. Additionally, these protein-rich matrices can promote plant growth during the degradation process, while their high-water retention capacity further enhances their functionality in agricultural applications [18].

CFRs supply a regular dose of micronutrients continuously, eliminating the need for multiple fertilizations. Thus, the problem of nutrient losses and root damage caused by excessive salt concentration is addressed [19]. The characteristics of these fertilizers must be the most appropriate for the nutritional needs of plants at a given stage of their growth [20]. The process of releasing nutrients from coated granules occurs in stages, beginning when the fertilizer is placed into the soil. The use of semi-permeable coatings or natural materials (such as protein-based) contributes to the controlled solubility of the fertilizer, increasing nutrient availability in the soil [21,22]. The rate of micronutrient release is influenced by two processes: diffusion through the pores and canals of a matrix and physical and biochemical degradation of the matrix [23]. Several thermomechanical methods, such as extrusion molding, injection molding and thermocompression, are employed to manufacture bioplastic matrices [24]. Among these, extrusion stands out due to its continuous nature and scalability, eliminating the need for a preliminary processing stage. Critical processing parameters, including plasticizer content, temperature, specific mechanical energy input (SME) and residence time, must be carefully optimized to ensure the fabrication of high-performance matrices [25].

During extrusion, an equilibrium is established between protein de-aggregation produced by mechanical stress and protein aggregation from heating [26]. Heat is delivered to the metering zone by both external heating devices and viscous dissipation. Excessive aggregation at high temperatures causes physical crosslinking, which reduces melt flow [27] and inhibits elastic recovery for large deformation. Optimal processing requires balancing the lower limit set by the glass transition temperature with the upper limit constrained by thermal degradation or excessive protein aggregation [28,29].

Moreover, the SME input measures the severity of extrusion conditions and is calculated using torque, screw speed and mass flow rate. It has a direct impact on the rheological properties of the melt, the extent of macromolecular transformations and additive interactions, resulting in polymers that could range from partially soluble to insoluble, enlarged or even degraded [30]. Thus, high SME and product temperatures lead to disrupted extrudates resulting from excessive crosslinking [31].

On the other hand, residence time and its distribution can have a significant impact on the characteristics of the bioplastics obtained. Thus, a high residence time, for example, can lead to thermal decomposition, but it could also be favorable if slow process kinetics are involved, such as the slow dissolution of a component in the melt. Low residence time, on the contrary, may result in inadequate dispersive and distributive mixing. This parameter is particularly relevant with respect to pharmacological formulations since it is highly related to the pharmaceutical effect as well as the toxicity of a formulation [32].

Finally, plasticizers are low-molecular-weight compounds that enhance flexibility and processability by disrupting hydrogen bonding and increasing macromolecular mobility, thereby reducing the glass transition temperature [33,34]. Beyond improving physical properties, plasticizers facilitate processing by reducing viscosity and required processing temperatures [35]. Water is a common plasticizer; however, due to its low boiling point, it tends to volatilize during the thermal process. Therefore, less volatile plasticizers with polar groups such as hydroxyl (-OH), carboxyl (-COOH) and amine (-NH_2_), for example, glycerol, are often preferred [36].

Despite extensive research on protein-based bioplastics, the combined, quantitative influence of the plasticizer mixture ratio (specifically water and glycerol) and extrusion temperature on the thermal stability, mechanical properties, water uptake behavior, microstructure, and micronutrient-release kinetics of SPI matrices remains insufficiently understood. This knowledge gap limits the ability to design protein-based systems with predictable performance for controlled nutrient delivery. Considering this, the present work aimed to develop soy protein-based bioplastic matrices incorporating iron as controlled-release fertilizers processed via extrusion molding. The influence of the type of plasticizer (water, glycerol and their mixtures at different ratios: 0:100, 25:75, 50:50, 75:25 and 100:0) and the processing temperature (70, 90 and 110 °C) on the physicochemical, mechanical, functional and microstructural properties of the bioplastic matrices was evaluated, as well as their effect on the iron release rate. The findings aim to inform the design of sustainable, biodegradable delivery systems with tailored nutrient-release profiles for precision agriculture applications. Accordingly, the central research question addressed in this study is how the combined variation in glycerol/water ratio and extrusion temperature governs the structural, mechanical and functional behavior of SPI-based matrices, ultimately determining their iron-release performance.

## 2. Materials and Methods

### 2.1. Materials

Soy Protein Isolate (SPI), a by-product of industrial soybean oil production, was used as the matrix material for the extruded systems. The SPI employed (SUPRO 500E, Protein Technologies International, Iepes, Belgium) consisted of approximately 91 wt% protein, with 5 wt% moisture and minor components. It was supplied as a yellowish powder with a particle size distribution of 100–240 µm. Glycerol (Gly, Escuder, Barcelona, Spain) and distilled water (W) were used as plasticizers to enhance the processability of the formulations. Iron sulfate heptahydrate (FeSO_4_·7H_2_O), obtained from Panreac Química S.A. (Barcelona, Spain), was incorporated as the iron-based micronutrient.

### 2.2. Preparation of the Bioplastic Matrices

The bioplastic matrices were prepared using the formulations listed in Table 1. These proportions employed are based on a previous study by Jiménez-Rosado et al. [37] in which it was demonstrated that the more micronutrients added, the more rigid and less deformable the matrices become, also significantly reducing their capacity to absorb water. To this end, the dry components (SPI and FeSO_4_·7H_2_O) were first mixed manually, followed by the separate mixing of the wet components (W and Gly). Both mixtures were combined and thoroughly homogenized prior to extrusion. The prepared mix was promptly loaded into a DSM Xplore mini twin-screw extruder (5 cc, Maastricht, The Netherlands), featuring a conical, fully intermeshing, corotating screw system with an L/D ratio of 8 and a compression ratio of 3.3. Extrusion was carried out through a circular die with a 2.8 mm diameter. The screw operated at 60 rpm, and all heating zones were maintained at 70 °C, 90 °C, or 110 °C. The samples were cut to 5 mm in length and 2.8 mm thick for subsequent tests.

### 2.3. Thermal Stability of Blends

The thermal properties of the formulations prior to extrusion were evaluated using differential scanning calorimetry (DSC). The analyses were conducted using a Mettler Toledo DSC 1 thermal analyzer (Mettler Toledo, Barcelona, Spain), with a 100 μL aluminum pan loaded with approximately 10 mg of sample. Measurements were taken over a temperature range of −10 to 250 °C, with a heating rate of 10 °C/min and a continuous nitrogen flow of 50 mL/min to prevent oxidation.

### 2.4. Characterization of the Bioplastic Matrices

#### 2.4.1. Thermal Stability of Bioplastic Matrices

The thermal stability of the bioplastic matrices was evaluated by thermogravimetric analysis (TGA) using a Mettler Toledo TGA/DSC 3+ (Mettler Toledo, Barcelona, Spain). Measurements were conducted over a temperature range of 30 to 800 °C, with a constant heating rate of 10 °C/min under a nitrogen atmosphere at a flow rate of 50 mL/min.

#### 2.4.2. Structural and Morphological Characterization

The structural characteristics of the bioplastics and their associated chemical bonds were analyzed using Fourier Transform Infrared Spectroscopy (FTIR) with a Hyperion 100 FTIR spectrometer (Bruker, Billerica, MA, USA) equipped with a diamond attenuated total reflection (ATR) sensor. Infrared spectra were recorded over 4000–600 cm^−1^ with a spectral resolution of 4 cm^−1^. Each measurement represented an average of 20 scans.

On the other hand, the cross-sectional internal microstructure of the extruded bioplastic matrices was examined using Scanning Electron Microscopy (SEM). For this purpose, the extruded samples were first immersed in liquid nitrogen for 5 min and subsequently cryo-fractured to expose their internal structure. To enhance surface conductivity and image resolution, the fractured surfaces were sputter-coated with Pt/Pd alloy using an Agar High-Resolution Sputter Coater (model 208RH, Leica Microsystems, Wetzlar, Germany) [38]. Finally, SEM imaging was performed using a Hitachi TM-1000 tabletop SEM (Hitachi High-Tech Group, Hitachinaka, Japan) operated at an accelerating voltage of 10 kV and a magnification of ×500.

#### 2.4.3. Mechanical Properties

Dynamic mechanical tests were performed in tension mode using a dynamic mechanical strain analyzer (RSA3, TA Instruments, New Castle, DE, USA). Firstly, the samples were subjected to a strain sweep test from 0.002% to 2% at a constant frequency of 1.0 Hz to determine the linear viscoelastic region, where the elastic and viscous moduli remain independent of strain. Subsequently, frequency sweep tests were conducted between 0.02 and 20 Hz within the established linear viscoelastic region.

#### 2.4.4. Water Uptake Capacity (WUC)

*WUC* tests were conducted in accordance with the ASTM D570-98 standard [39]. In these tests, the bioplastic matrices were immersed in 300 mL of distilled water for 24 h. Thus, *WUC* was calculated following Equation (1):

(1) $$WUC\left(\%\right)=\frac{m_{2}-m_{3}}{m_{3}}\cdot 100$$

where *m*_2_ is the weight of the sample after water immersion and *m*_3_ is the weight of the dry sample after the *WUC* test.

#### 2.4.5. Iron Release in Water

The micronutrient release from the matrices was controlled using a method adapted from Cong et al. [40] and Essawy et al. [41]. To evaluate iron release, the bioplastic matrices were immersed in 100 mL of distilled water, and the electrical conductivity of the solution was monitored over time using an EC-meter (Mettler Toledo, Barcelona, Spain). As conductivity is directly related to the dissolution of ionic species, the micronutrient release was considered complete when the conductivity readings stabilized for more than one hour.

#### 2.4.6. Mold Resistance

A mold resistance test was conducted on the extruded samples to assess their susceptibility to fungal growth under specific temperature and humidity conditions that replicate extreme storage scenarios during shelf life [42]. For this purpose, 0.5 g of each sample was placed in separate wells of cell culture plates, which were then stored in a sealed container with Milli-Q water at the bottom to maintain a relative humidity of 100%. The setup was kept at 25 °C and exposed to natural light. To closely mimic real-world storage conditions, the samples were tested without prior sterilization or intentional fungal inoculation. Mold development was monitored through weekly photographic reports over a period of three weeks.

### 2.5. Statistical Analysis

At least three replicates of each measurement were made. Statistical analyses were performed using one-way analysis of variance (ANOVA *p* < 0.05) followed by Tukey’s post hoc test. The mean and standard deviation of each measurement were calculated.

## 3. Results and Discussion

### 3.1. Thermal Stability of Blends

**Figure 1:**
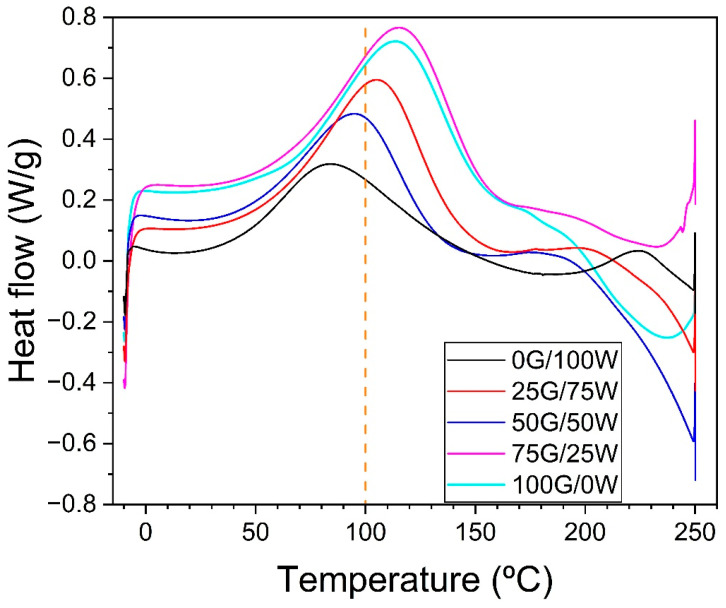
Differential Scanning Calorimetry (DSC) analysis of pre-extrusion blends with varying plasticizer ratios. G: Glycerol; W: Water.

Figure 1 shows the DSC thermal profile of the blends with different formulations. A broad endothermic peak was observed between approximately 55 °C and 150 °C across all formulations. This thermal event is the result of multiple overlapping phenomena. These include the evaporation of structurally bound water, enthalpic relaxation and progressive thermal plasticization of the matrix [43]. Notably, formulations with higher glycerol content exhibited a shift in this peak toward higher temperatures, from 75 °C for 0G/100W to approximately 120 °C for 100G/0W, suggesting that glycerol stabilizes water–polymer interactions and restricts molecular mobility within the matrix. This suggests that glycerol not only acts as a plasticizer but also modifies the thermal response of the system by reinforcing hydrogen-bonding networks and modulating water retention dynamics within the matrix.

No additional thermal transitions or decomposition peaks were detected up to 250 °C in any of the tested formulations. This result indicates that the biopolymeric matrices, regardless of the glycerol-to-water ratio, exhibit considerable thermal stability within this extended temperature range. The absence of significant exothermic or endothermic signals above 150 °C suggests that the materials do not undergo further structural rearrangements, melting transitions or degradation phenomena within the operational range typically used in extrusion processes [44]. This is particularly relevant for thermal processing, as it confirms that the formulations can be heated safely beyond the main thermal transition without risk of early decomposition or undesirable physicochemical changes. From a processing standpoint, this thermal resilience provides a wide safety margin for setting extrusion temperatures [45]. It ensures that the material remains stable under the thermal and mechanical stresses applied in the extruder, especially in zones where temperatures may exceed 150 °C. Furthermore, this stability also reflects the effectiveness of glycerol as a plasticizer, not only in modifying the thermal transitions at lower temperatures but also in maintaining the overall integrity of the matrix structure at elevated temperatures [46].

### 3.2. Characterization of the Bioplastic Matrices

#### 3.2.1. Thermal Stability of Bioplastic Matrices

**Figure 2:**
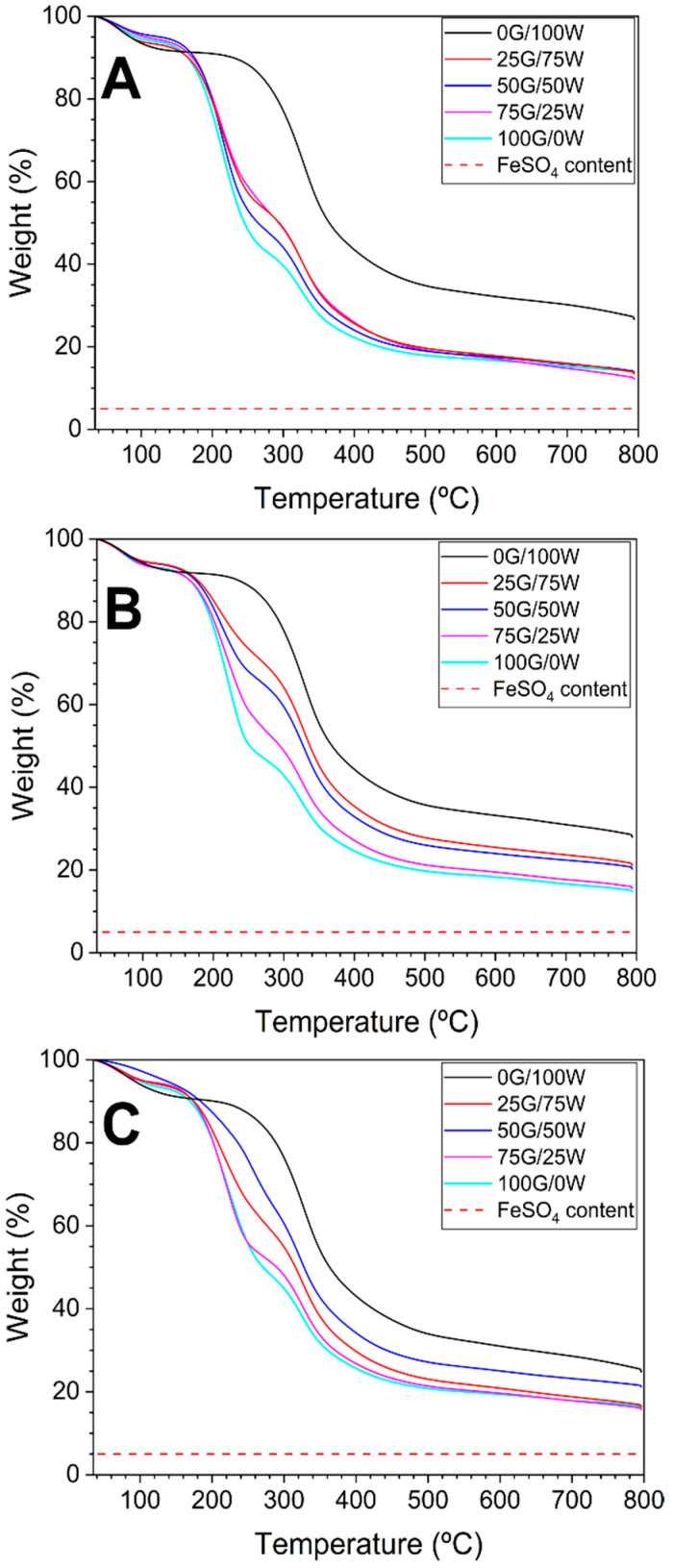
Thermogravimetric analysis (TGA) of bioplastic matrices processed at 70 (**A**), 90 (**B**) and 110 °C (**C**). G: Glycerol; W: Water. The dashed lines refer to FeSO_4_.

Thermogravimetric analysis of the bioplastic matrices (Figure 2) shows that most samples exhibit a characteristic three-step weight-loss profile. The initial weight loss of about 5 to 10 wt% (below 100 °C) corresponds to the evaporation of free or weakly bound water, followed by a second step (100–150 °C) in which another 5 wt% is lost, associated with volatile components, such as glycerol [47,48]. Finally, the main degradation phase (200–400 °C) corresponds to the thermal breakdown of the polymer matrix, in which around 40–60 wt% is lost [49]. Figures S1–S3 show the derivative signals from the TGA analyses of the bioplastic matrices processed at 70, 90, and 110 °C, respectively, facilitating easier appreciation of the degradation steps mentioned above.

Importantly, an increase in glycerol content led to an earlier onset of protein degradation at about 10 °C, for example, between system 0G/100W which seemed to degrade around 320 °C, and system 100G/0W which degraded at 310 °C, suggesting that glycerol, while acting as an effective plasticizer, also facilitates thermal rearrangement or relaxation of the protein network, thereby reducing the thermal stability of the matrix. Processing temperature also had a clear impact [50]. At 70 °C, the high moisture retention led to broader, less distinct degradation steps, indicating incomplete drying and structural heterogeneity. At 90 °C, samples exhibited reduced water loss and more defined thermal transitions, indicating that this temperature offers optimal processing by balancing dehydration and matrix integrity. At 110 °C, although moisture was nearly eliminated, early degradation onset was observed in glycerol-rich formulations, aligning with DSC results, which suggest that an elevated glycerol content promotes internal thermal rearrangements that compromise the thermal stability of the protein network.

#### 3.2.2. Structural and Morphological Characterization

**Figure 3:**
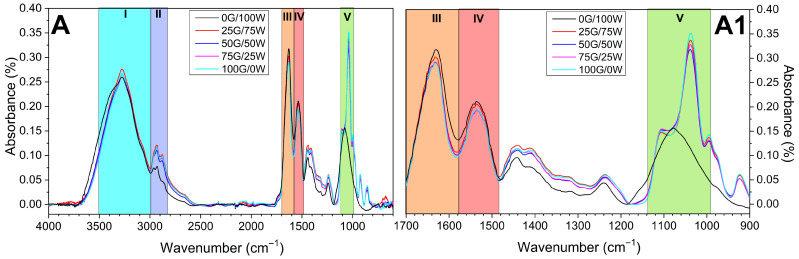

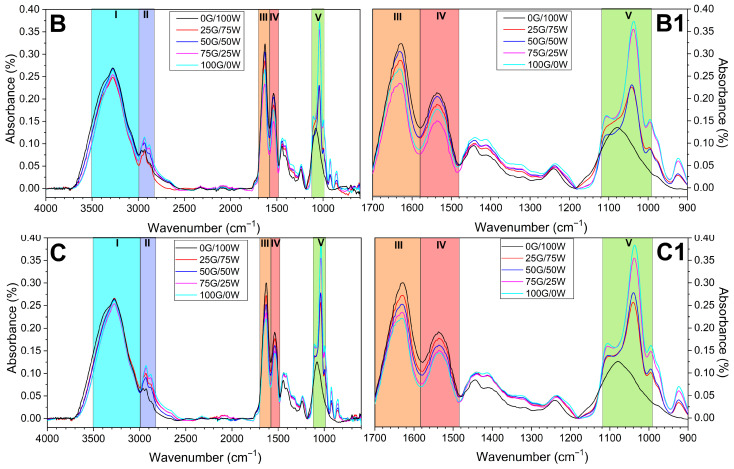
FTIR profiles of the bioplastic matrices processed at 70 (**A**), 90 (**B**) and 110 °C (**C**) and the amplification of zones III, IV and V between 1700 and 900 cm^−1^ ((**A1**), (**B1**) and (**C1**), respectively). G: Glycerol; W: Water.

Figure 3 presents the FTIR spectra of the formulated systems. All samples exhibited comparable spectral profiles, indicating similar functional group distributions across the formulations. A broad absorption band between 3500 and 3000 cm^−1^ (region I) corresponds to O-H stretching, N-H stretching (associated with amide A and B) and the harmonic vibration of the amide II band [51]. The absorption bands at 2925 and 2871 cm^−1^ (region II) are attributed to the stretching vibrations of CH and CH_2_ groups, characteristic of protein backbone structures [51]. The most relevant features appear at 1625, 1544 and 1227 cm^−1^ (regions III, IV and V, respectively), corresponding to the amide I, II and III bands, respectively, associated with C=O stretching, N–H bending, and C–N stretching vibrations [52]. Deconvolution of the Amide I band (1600–1700 cm^−1^) revealed the typical contributions of β-sheet (1610–1630 cm^−1^), random coil (1635–1650 cm^−1^), α-helix (1650–1660 cm^−1^) and β-turn (1660–1680 cm^−1^) structures. The spectra show that glycerol addition induces modifications in both the Amide I region and the broad O–H stretching band, evidencing the formation of protein–glycerol hydrogen bonds. These characteristic peaks are indicative of the protein’s secondary structure. Notably, as the processing temperature increased, the intensity of these bands decreased progressively. This attenuation is indicative of chemical alteration within the protein network, likely due to heat-induced conformational changes. Similar trends have been reported in the literature and are commonly associated with enhanced protein aggregation and greater degrees of denaturation, which result from thermal processing [10]. The reduction in amide band intensity thus reflects a partial loss of the ordered protein secondary structures, consistent with heat-induced conformational rearrangements within the protein matrix.

In all formulations, the amide I and amide II regions showed that glycerol content clearly influenced the secondary structure of SPI. Thus, SPI globulins (glycinin 11S and β-conglycinin 7S) exhibit a mixture of α-helix, β-sheet, β-turn and random coil conformations, forming a network stabilized by intramolecular hydrogen bonds and disulfide bridges. Upon film formation, partial unfolding and aggregation increase the β-sheet content and protein–protein interactions. Glycerol molecules interact mainly via hydrogen bonding with the peptide backbone and polar side chains, partially disrupting protein–protein hydrogen bonds and forming protein–glycerol–protein bridges. This leads to increased chain mobility and a more flexible, less brittle protein network [53].

**Figure 4:**
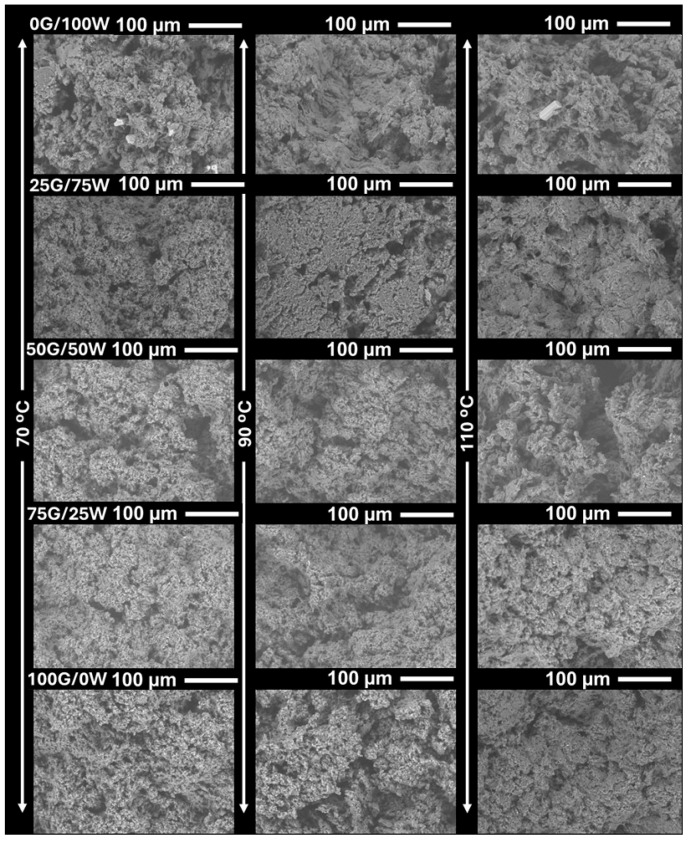
Scanning electron micrographs of the biopolymeric matrices prepared with different glycerol(G)/water(W) ratios (0G/100W to 100G/0W) and processing at 70, 90 and 110 °C.

Figure 4 displays SEM micrographs of biopolymeric matrices with varying glycerol-to-water ratios and processed at 70, 90, and 110 °C. The samples treated at 70 °C exhibited porous and irregular structures, particularly in glycerol-deficient formulations, suggesting insufficient water removal and the presence of collapse-prone domains. In contrast, samples extruded at 90 °C showed a more compact, homogeneous structure across all compositions, indicating optimal moisture removal and minimal thermal degradation. Samples processed at 110 °C exhibited slightly denser, more granular surfaces, particularly in water-rich formulations, likely due to thermally induced contraction or microstructural rearrangements during rapid moisture loss. This behavior can be rationalized by a mechanism akin to that described by Meng et al. [54], in which water acts simultaneously as a plasticizer and a blowing agent. Thus, vaporization of water during extrusion induced bubble formation whose growth and stabilization depended on the moisture content and thermal profile. Such behavior may explain the more open and irregular morphology observed in water-rich matrices at lower temperatures. The progressive increase in glycerol content also led to more cohesive, continuous microstructures, consistent with its plasticizing and stabilizing effects on the polymeric matrix. These microstructural observations align well with the DSC and TGA data, supporting the conclusion that both composition and processing temperature critically affect the structural integrity and uniformity of the final bioplastic material.

#### 3.2.3. Mechanical Properties

**Figure 5:**
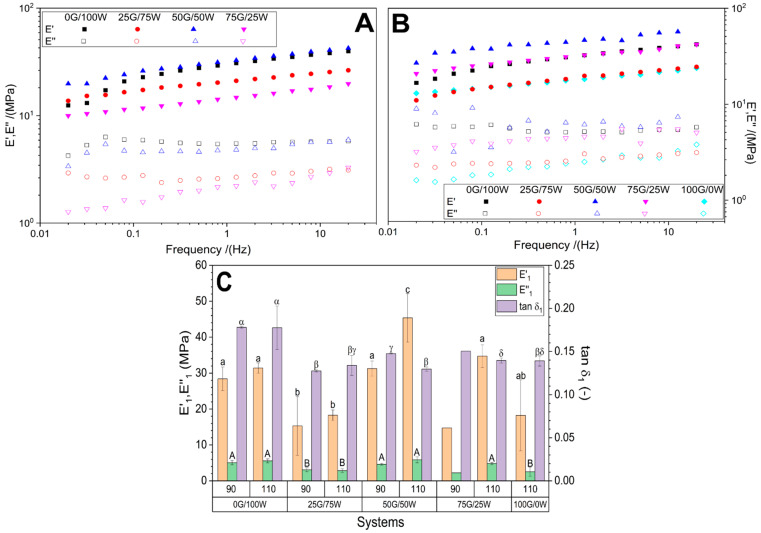
Dynamic mechanical analysis (DMA) of bioplastic matrices processed at 90 °C (**A**) and 110 °C (**B**). Storage modulus (E’) and loss modulus (E’’) are plotted as a function of frequency for each composition. (**C**) Storage modulus, loss modulus and loss tangent values at 1 Hz (E’_1_, E’’_1_ and tan δ_1,_ respectively). a, b, c and ab indicate significant differences (*p* < 0.05) in E’_1_ in different groups. A and B indicate significant differences (*p* < 0.05) in E’’_1_ in different groups. α, β, γ and δ indicate significant differences (*p* < 0.05) in tan δ_1_ in different groups.

Figure 5 shows the frequency dependence of the storage modulus (E’) and loss modulus (E’’) for the bioplastic formulations processed at 90 °C (A) and 110 °C (B). Moreover, in Figure 5C, storage modulus, loss modulus, and loss tangent values at 1.0 Hz are represented for a better comparison. The samples conditioned at 70 °C were too brittle to withstand the dynamic mechanical analysis, consistent with previous thermal and structural findings.

At both processing temperatures, all samples exhibited the expected increase in E’ and E’’ with frequency, reflecting viscoelastic behavior. Among the tested formulations, the 50G/50W formulation consistently displayed the highest E’ value across all frequencies, outperforming other formulations and indicating superior stiffness and elastic energy storage capacity, as can be confirmed by observing Figure 5C comparing E’_1_ since the intermediate system exhibited a value of around 32 MPa at 90 °C or 44 MPa at 110 °C, while the next highest at 90 °C was 0G/100W with a value of around 28 MPa and 75G/25W at 110 °C with a value of 34 MPa. At 110 °C, a general increase in modulus was observed for most formulations, particularly 50G/50W and 75G/25W, which increased from 32 and 14 MPa to 44 and 34 MPa, respectively, suggesting enhanced molecular packing or structural densification, likely due to thermal reorganization. In contrast, the 100G/0W sample (measured only at 110 °C, as it was too brittle at 90 °C) exhibited the lowest moduli, confirming the pronounced plasticizing effect of glycerol. These results reinforce the critical influence of both composition and processing temperature on the mechanical performance of the biopolymeric matrices, with 50G/50W emerging as the formulation with the most favorable balance between rigidity and viscoelasticity. A Tukey post hoc test (*p* < 0.05) revealed statistically significant differences in E’_1_ values between systems with different plasticizer ratios. Moreover, no significant differences were observed when varying the temperature in systems 0G/100W and 25G/75W. However, when higher glycerol proportions are used, significant differences within the same systems are observed. In this sense, the system with an intermediate concentration of both plasticizers (50G/50W) differs significantly from the other systems, exhibiting the highest E’_1_ value at 100 °C due to an enhanced molecular packing as mentioned above.

#### 3.2.4. WUC

**Figure 6:**
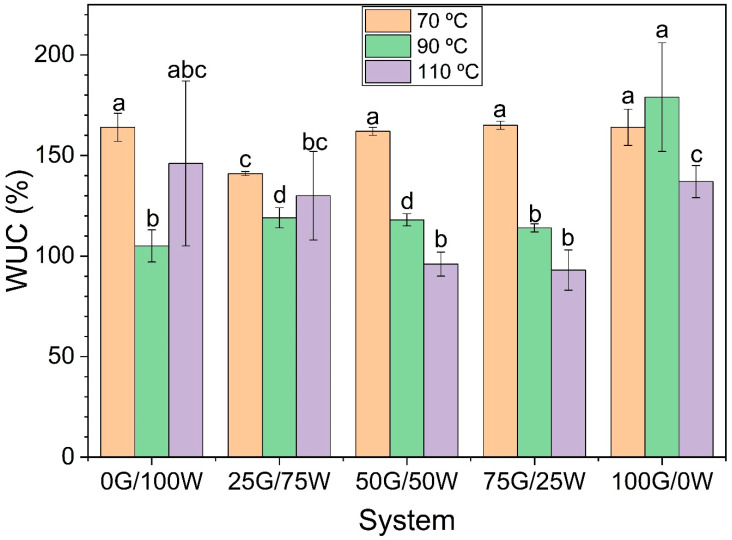
Water Uptake Capacity of the bioplastic matrices made with different amounts of water and processing temperatures. a, b, c, d, bc and abc indicate significant differences (*p* < 0.05) in Water Uptake Capacity (WUC) values in different groups.

Figure 6 shows the water uptake capacity (WUC) of the biopolymeric formulations as a function of initial water content and processing temperature (70, 90 and 110 °C). In general, thermal treatment influenced WUC, but the effect was dependent on the glycerol-to-water ratio.

As observed, no significant differences are generally found between the systems when comparing the WUC values at the same temperatures. Nonetheless, when varying the extrusion temperature, some differences can be observed within the systems. Thus, for formulations with intermediate water contents (25–75%), a clear decrease in WUC was observed as the processing temperature increased from an average of 170% at 70 °C to 100% at 110 °C for systems 50G/50W and 75G/25W. This behavior suggests that higher drying temperatures promote matrix densification, thereby limiting the availability of hydrophilic domains for water absorption. However, at the extremes of composition, particularly 0 and 100% water, this trend did not hold. Notably, the 100G/0W formulation (100% glycerol) exhibited a slight increase in WUC at 90 °C compared to 70 °C, followed by a significant decrease at 110 °C. This suggests that moderate thermal conditioning may enhance the matrix’s capacity to absorb water, potentially due to structural relaxation or reorganization that exposes more hydrophilic sites.

In contrast, the 0G/100W formulation exhibited an opposite pattern: a decrease in WUC from 70 °C to 90 °C, suggesting increased densification, followed by partial recovery at 110 °C. This rebound likely results from thermal degradation or microstructural loosening at elevated temperatures, increasing water accessibility despite the absence of water in the original formulation. These findings highlight a nonlinear relationship between composition, thermal processing and water uptake, confirming that both water and glycerol content modulate the plasticization and structuring processes during drying. The results are consistent with the morphological differences observed by SEM and reinforce the need to optimize thermal processing parameters for each formulation.

#### 3.2.5. Iron Release in Water

**Figure 7:**
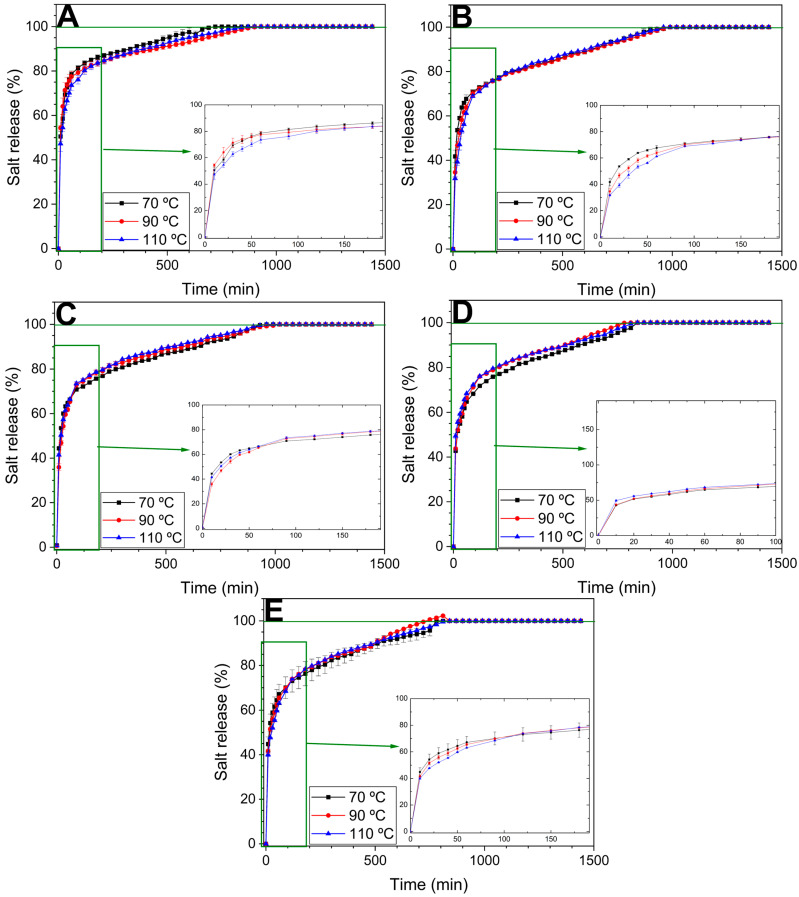
Kinetics of iron release in water from different bioplastic matrices processed at 70, 90, and 110 °C with varying glycerol (G)/water (W) ratios. (**A**): 0G/100W. (**B**): 25G/75W. (**C**): 50G/50W. (**D**): 75G/25W. (**E**): 100G/0W.

Figure 7 shows the iron release profiles in water for the different bioplastic formulations subjected to thermal processing at 70, 90, and 110 °C. All samples demonstrated rapid initial release, followed by a plateau phase, reaching nearly complete release (defined as the point at which conductivity remained stable for over 1 h, indicating >95% of the iron has diffused into the medium) within 500 min. The effect of drying temperature was minimal overall, although minor differences in release rate were observed during the early stages, suggesting that temperature may influence matrix permeability or initial surface dissolution dynamics.

The 0G/100W formulation exhibited the fastest release, likely due to its highly porous and hydrophilic structure. In contrast, the 100G/0W formulation showed the slowest initial release, attributed to its denser, glycerol-rich matrix, which limited water penetration and ion diffusion. Intermediate formulations (25G/75W to 75G/25W) exhibited consistent release profiles across all processing temperatures, indicating a balanced interplay between matrix permeability and structural integrity. These differences can be better appreciated in Table 2, which presents the average release rate. Thus, as mentioned before, the system 0G/100W exhibited by far, the highest average release rate of iron and while, the opposite system (100G/0W) exhibited an initial slower release, as seen in Figure 7E and confirmed by the data in Table 2, the rate of release increased with time, reaching release rates of 7.1 to 7.7% of iron released per hour. Intermediate systems (25G/75W and 50G/50W) generally showed a more sustained release, with lower average release rates. 75G/25G showed a higher rate, but Figure 7D suggests this is mainly due to a high initial release speed, as at intermediate times a more sustained release is observed. These results confirm that the matrix composition exerts a greater influence on iron release dynamics than the drying temperature. This behavior aligns with that reported by Duan et al. [55]. According to their analyses, release from polymeric matrices is controlled by a coupled process: water penetration into the matrix, leading to hydration and swelling, followed by nutrient dissolution and its subsequent diffusion through the hydrated network. Thus, systems containing glycerol tended to form denser structures, resulting in slower, sustained iron release. Higher glycerol content appears to enable a more sustained or controlled release profile, highlighting its potential for applications requiring prolonged micronutrient delivery.

#### 3.2.6. Mold Resistance

**Figure 8:**
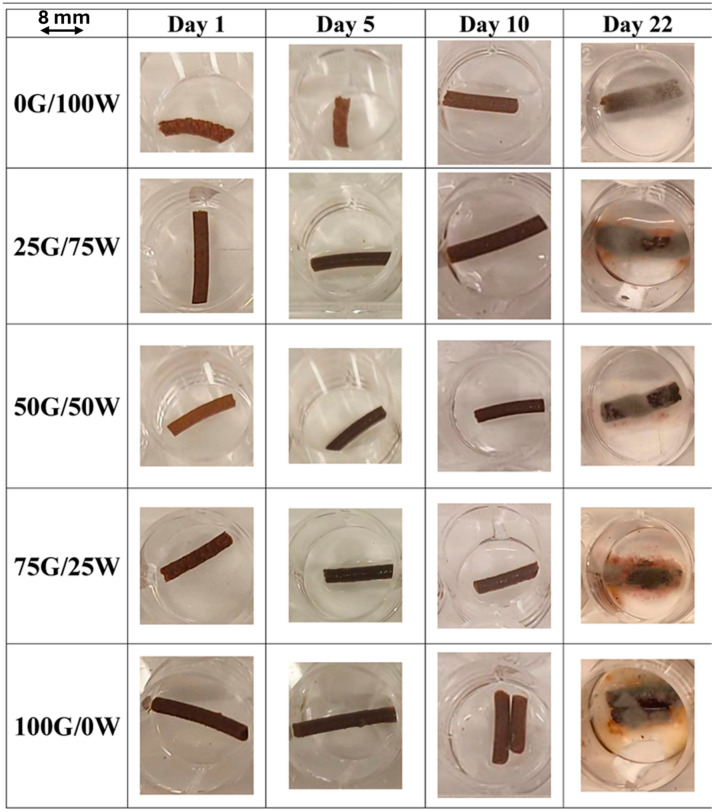
Mold resistance evaluation of bioplastic matrices processed at 90 °C and at different glycerol(G)/water(W) ratios.

Figure 8 displays the visual evolution of fungal growth on the different bioplastic formulations processed at 90 °C, monitored over 22 days under high humidity conditions. All samples eventually exhibited signs of mold colonization, although the onset, extent and intensity of fungal development varied, clearly influenced by the formulation’s composition.

The 100G/0W formulation was the most susceptible, showing visible fungal growth by day 22. This behavior may be attributed to glycerol’s hygroscopic nature, which can retain environmental moisture and create conditions favorable to microbial proliferation. In contrast, the 0G/100W formulation showed minimal visible fungal colonization, suggesting that a water-rich matrix without a plasticizer may dry more effectively on the surface or present fewer nutrients accessible to fungal growth. Intermediate formulations exhibited moderate growth, suggesting that fungal resistance is not linearly dependent on water or glycerol content, but rather a complex result of surface moisture retention, nutrient availability, and microstructure. These observations confirm that although glycerol can promote initial fungal proliferation, none of the formulations demonstrated complete resistance under prolonged exposure to high humidity. Therefore, optimizing both the matrix composition and packaging conditions will be essential for applications requiring extended shelf life. Additionally, the matrices processed at 70 and 110 °C display similar fungal growth patterns (see Supplementary Information, Figures S5 and S6, respectively), indicating that the processing temperature has a limited effect on mold resistance.

## 4. Conclusions

This study successfully developed soy protein-based bioplastic matrices containing iron as a micronutrient for controlled-release applications, using extrusion molding as a scalable and sustainable processing method. The work comprehensively investigated how the plasticizer composition (glycerol/water ratio) and processing temperature (70, 90, and 110 °C) influence the physicochemical, mechanical, functional, and structural characteristics of the resulting materials.

The nature and ratio of the plasticizer emerged as key factors that modulate the matrix structure and performance. Glycerol, due to its hygroscopic and hydrogen-bonding properties, enhanced cohesion, flexibility, and plasticization efficiency, whereas water primarily acted as a porogen, promoting porosity and rapid hydration. The interplay between these two components determined the mechanical, thermal, and functional behavior of the final bioplastic matrices. Intermediate formulations, particularly the 50G/50W system, exhibited the best balance between mechanical strength, thermal stability and water interaction properties. On the other hand, processing temperature significantly influenced material properties, with 90 °C emerging as the optimal temperature, referring to the formulation–temperature combination that allows for a synergy between high mechanical resistance, moderate WUC values, and a sustained micronutrient release.

Overall, this work highlights the potential of soy protein-based systems as customizable platforms for nutrient delivery, where composition and thermal processing must be carefully tuned to meet the demands of controlled-release performance, material stability and resistance under realistic storage conditions. This research demonstrates how sustainable materials can be engineered for functional performance without compromising ecological principles, a step forward in transforming renewable resources into smart delivery systems with real-world impact.

Future work should explore strategies to quantify mold growth, integrate microscopy observations to enhance microbial resistance, such as incorporating natural antifungal agents or surface coatings, and assess long-term stability under varied storage conditions. Additionally, the scalability and environmental impact of the materials should be evaluated in real-world settings to facilitate their industrial adoption.

## Figures and Tables

**Table 1:** Composition of the formulations used for the preparation of bioplastic matrices.

System	SPI (wt%)	FeSO_4_·7H_2_O (wt%)	W (wt%)	Gly (wt%)
100G/0W	47.50	5.00	0	47.50
75G/25W			35.63	11.87
50G/50W			23.75	23.75
25G/75W			11.87	35.63
0G/100W			47.50	0

**Table 2:** Average iron release rate (in % of iron released/h) in water of the bioplastics matrices processed at 70, 90 and 110 °C with varying glycerol/Water ratios.

	70 °C	90 °C	110 °C
0G/100W	8.3	6.5	6.9
25G/75W	6.1	6.1	6.3
50G/50W	6.3	5.9	6.3
75G/25W	7.1	7.4	7.1
100G/0W	7.7	7.1	7.1

## Data Availability

Data will be made available on request.

## Supplementary Materials

The following supporting information can be downloaded at: https://www.mdpi.com/article/10.3390/polym17233209/s1. Figure S1: Derivative signals of the thermogravimetric curves of the bioplastics matrices processed at 70 °C; Figure S2: Derivative signals of the thermogravimetric curves of the bioplastics matrices processed at 90 °C; Figure S3: Derivative signals of the thermogravimetric curves of the bioplastics matrices processed at 110 °C; Figure S4: Visual scheme of the molecular interactions between the backbone of the secondary structure of SPI and glycerol; Figure S5: Mold resistance evaluation of bioplastic matrices processed at 70 °C and at different glycerol(G)/water(W) ratios; Figure S6: Mold resistance evaluation of bioplastic matrices processed at 110 °C and at different glycerol(G)/water(W) ratios.
