# Supplementary figures and images for: Tailoring the Properties of Soy Protein-Based Bioplastics via Plasticizer Composition and Extrusion Temperature for Controlled Iron Release
Source: Polymers (Basel). 2025 Dec 2;17(23):3209. doi: 10.3390/polym17233209 (PMC12694450; doi:10.3390/polym17233209)

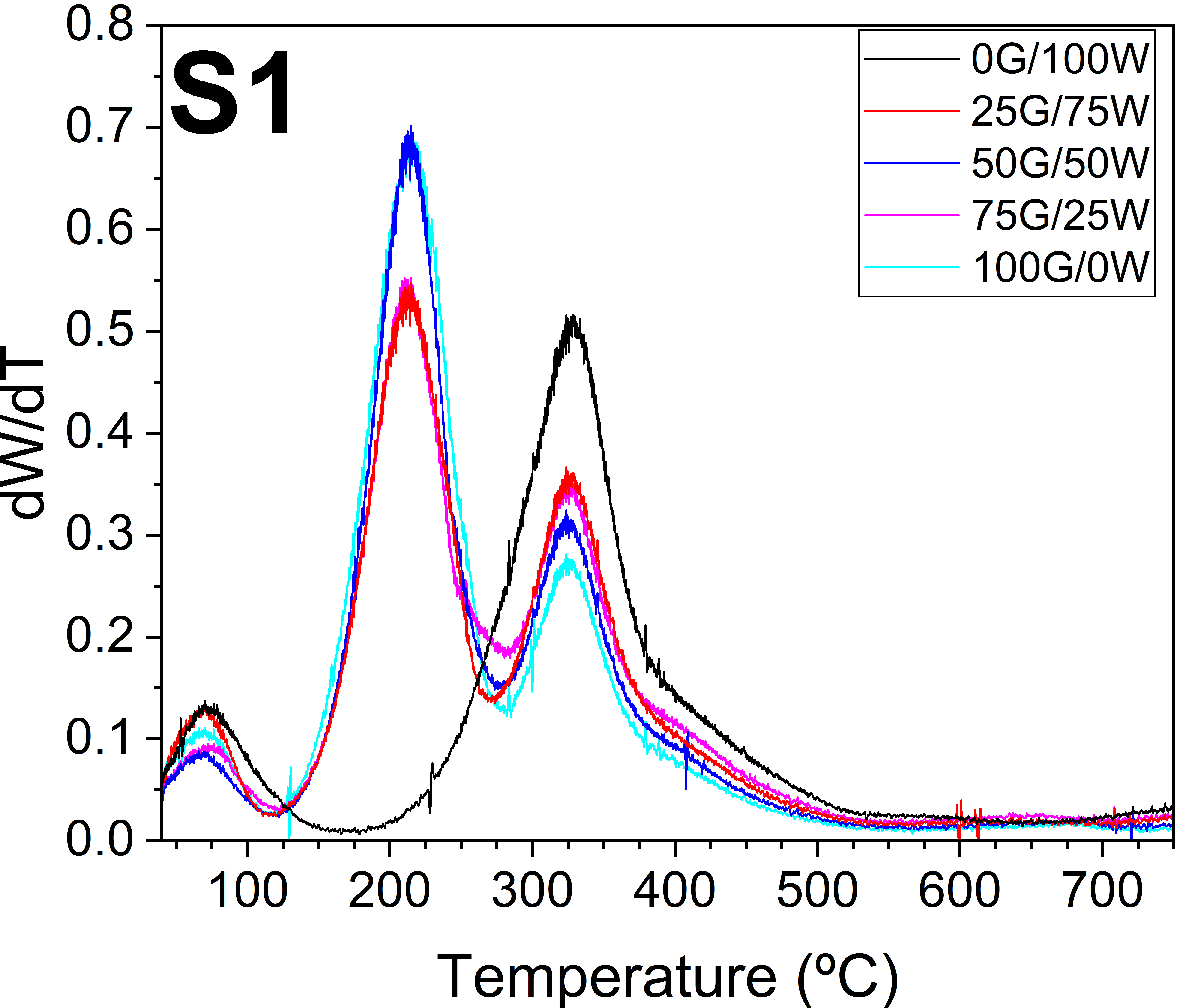

Supplement: Supplementary file 1 [file polymers-17-03209-s001.zip › Figure S1.Derivative TGA 70.tif]

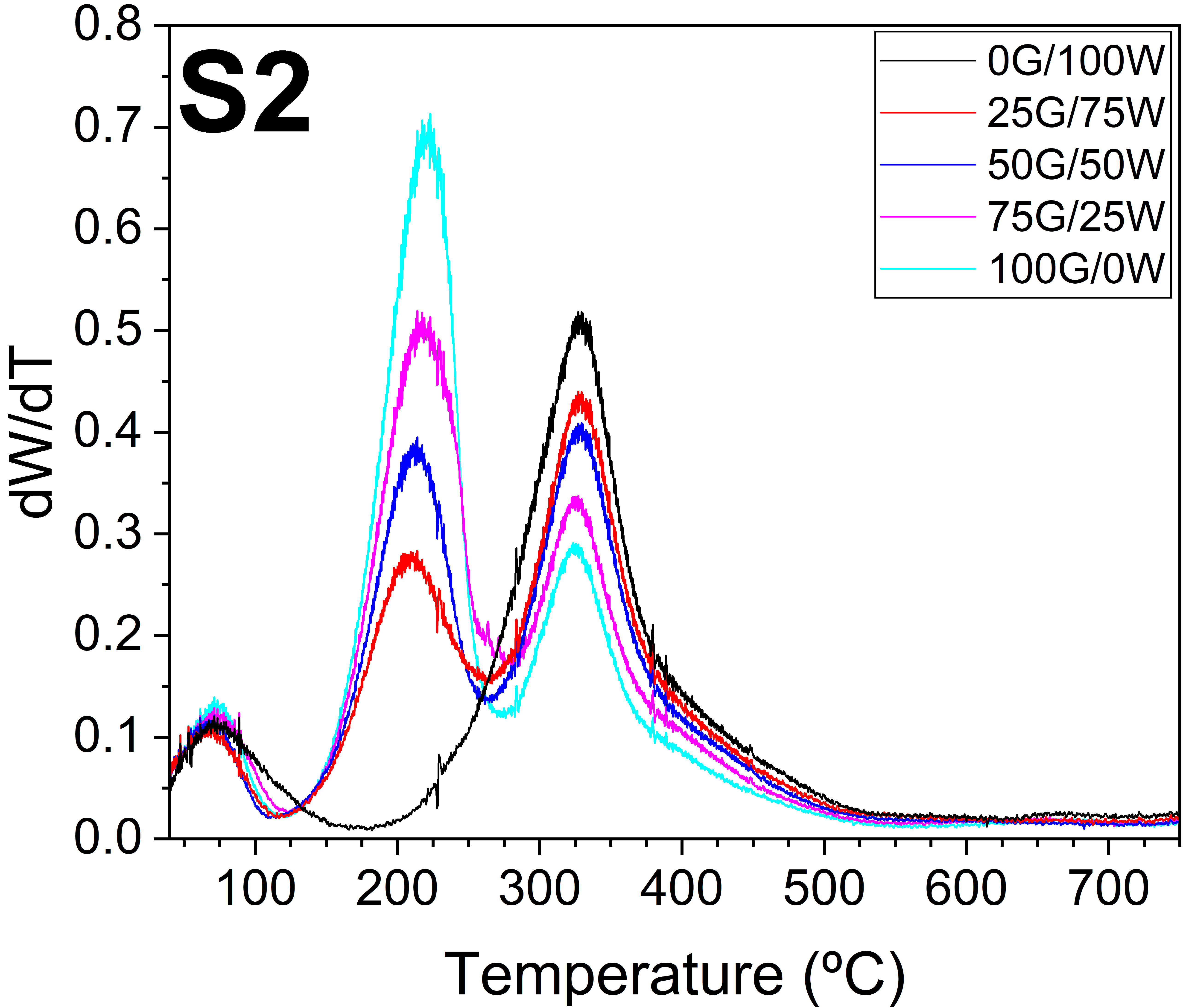

Supplement: Supplementary file 1 [file polymers-17-03209-s001.zip › Figure S2.Derivative TGA 90.tif]

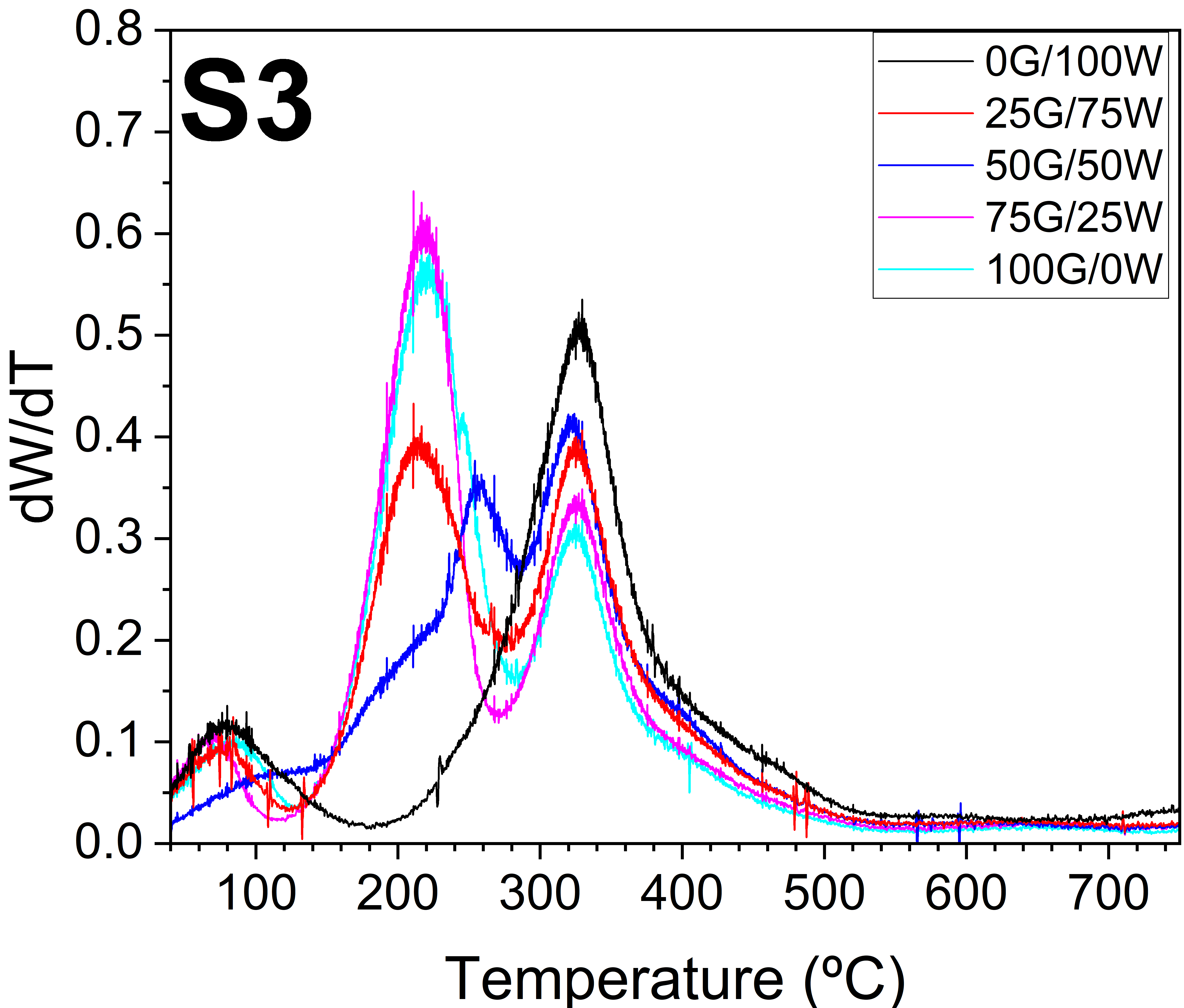

Supplement: Supplementary file 1 [file polymers-17-03209-s001.zip › Figure S3.Derivative TGA 110.tif]

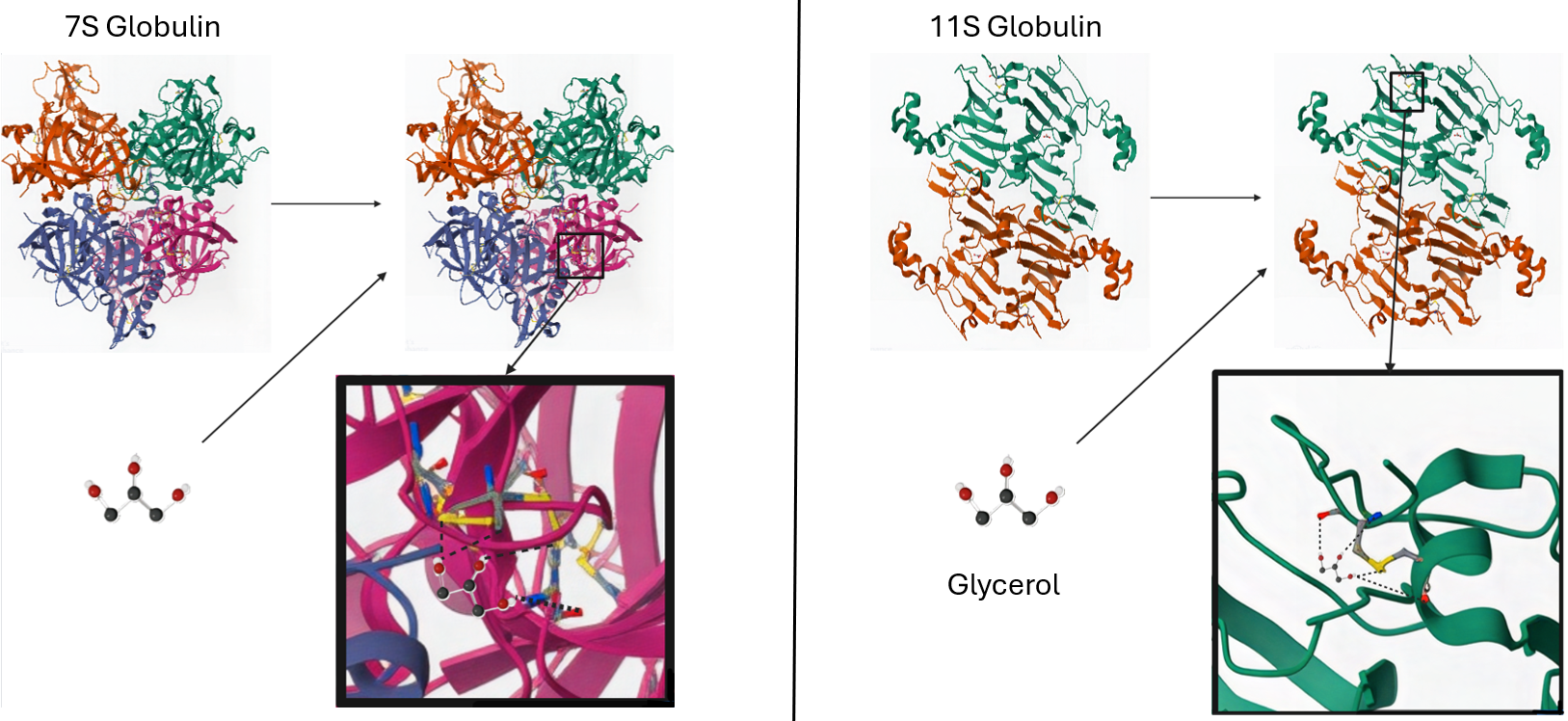

Supplement: Supplementary file 1 [file polymers-17-03209-s001.zip › Figure S4. Visual scheme of the molecular interactions between SPI and glycerol.png]

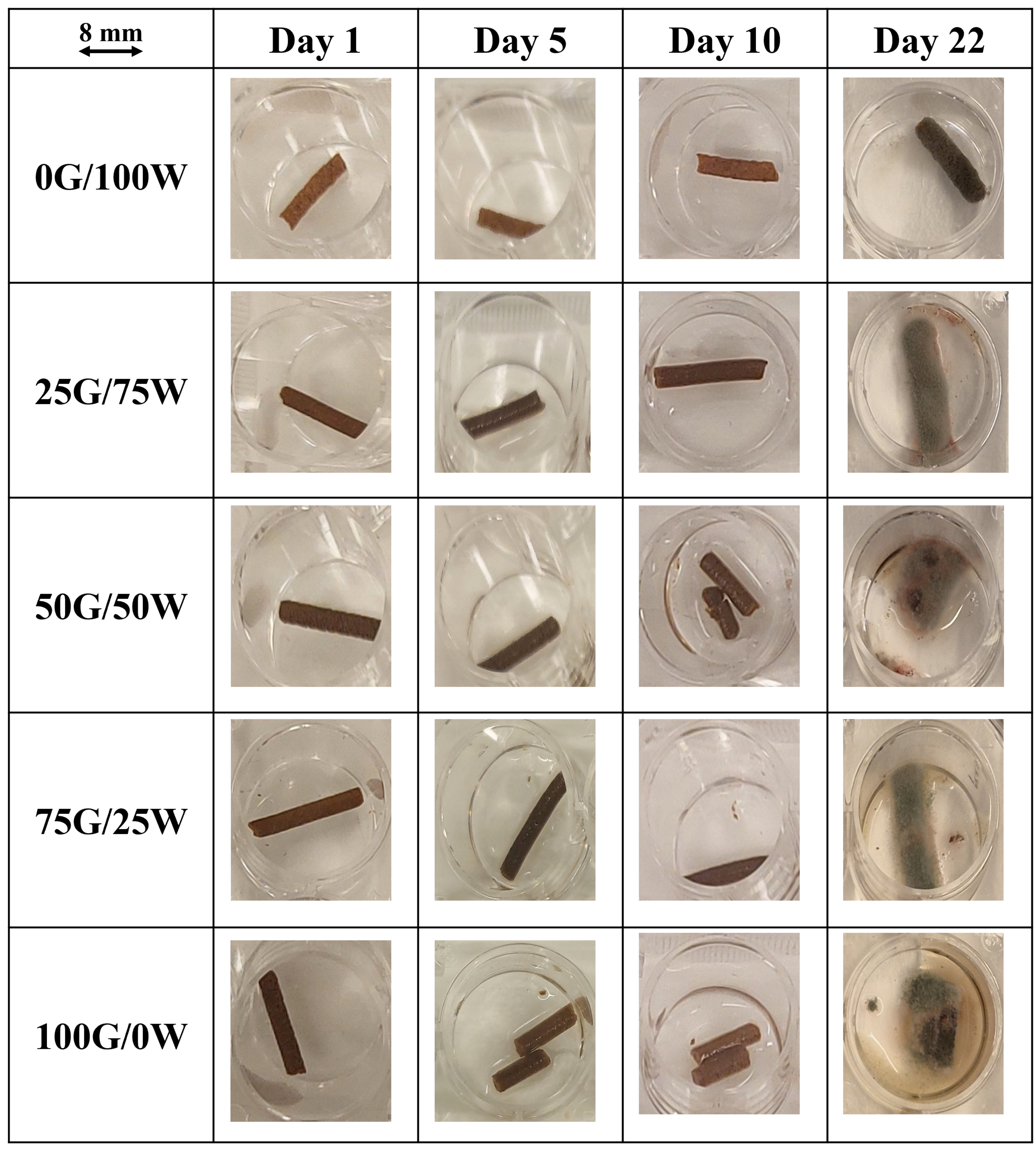

Supplement: Supplementary file 1 [file polymers-17-03209-s001.zip › Figure S5. Mold resistance evaluation of bioplastic matrices processed at 70 oC and at different glycerol(G)-water(W) ratios.tif]

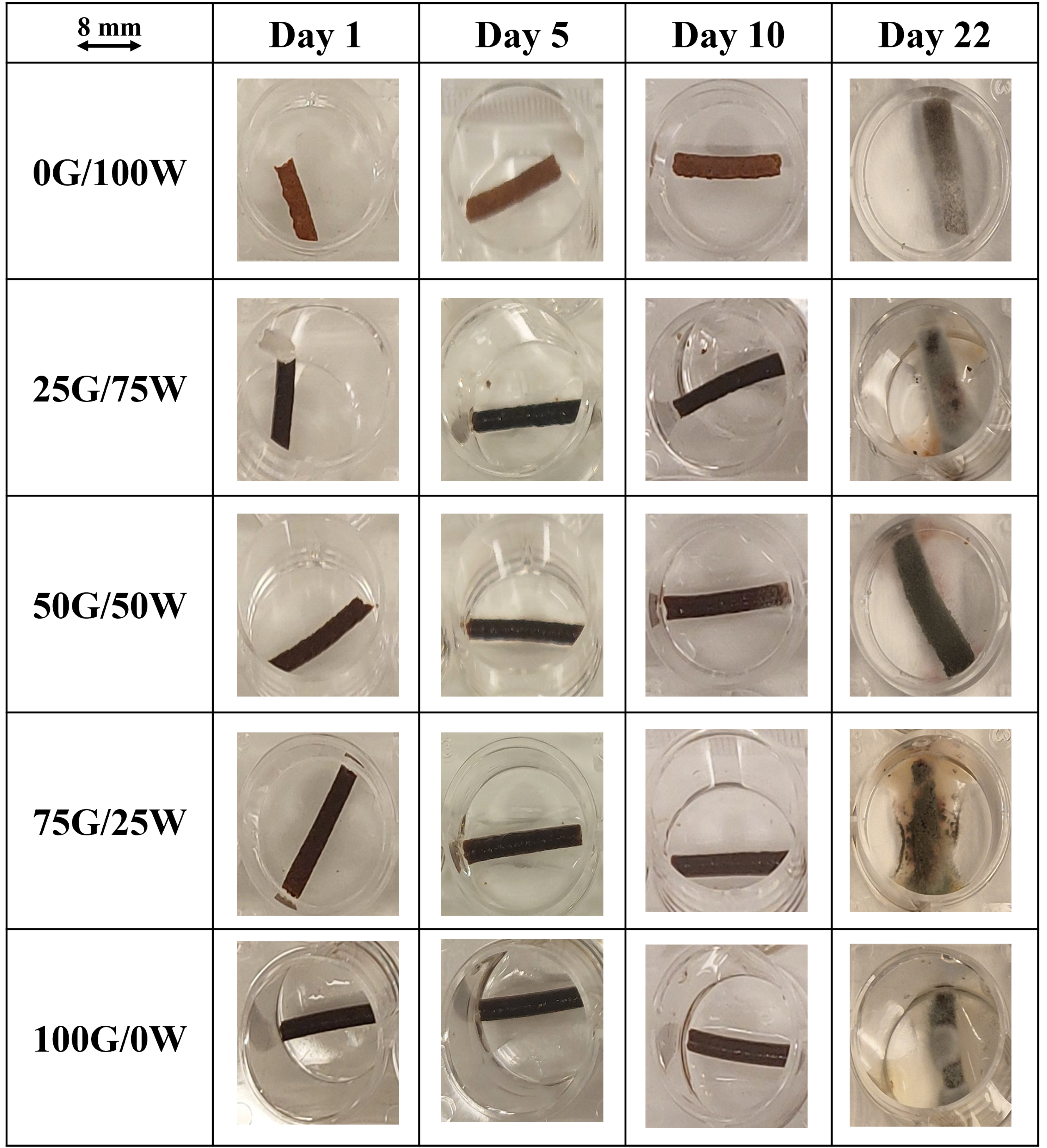

Supplement: Supplementary file 1 [file polymers-17-03209-s001.zip › Figure S6. Mold resistance evaluation of bioplastic matrices processed at 110 oC and at different glycerol(G)-water(W) ratios.tif]
